# Seat belt syndrome is caused by a different mechanism: A case report

**DOI:** 10.1016/j.ijscr.2022.107509

**Published:** 2022-08-13

**Authors:** R.M.G.K. Rathnayaka, Kalaventhan Pathinathan, S. Sivamynthan, Romesh Madushanger, Parathan Sriharan, Dilshan Munidasa

**Affiliations:** a Postgraduate trainee in General Surgery, Postgraduate Institute of Medicine, University of Colombo, Sri Lanka; b Postgraduate trainee in Orthopaedic Surgery, Postgraduate Institute of Medicine, University of Colombo, Sri Lanka; c Consultant Orthopedic Surgeon and Clinical Lead, Department of Trauma and Orthopaedics, National Hospital-Colombo, Sri Lanka.

**Keywords:** Seat belt syndrome, Intestinal perforation, Thoraco-lumbar fracture, Laparotomy, Case report

## Abstract

**Introduction and importance:**

Seat belt syndrome is associated with multiple visceral injuries and vertebral burst fractures. Small Intestinal perforations are seen in 5–15 % of blunt abdominal trauma. In our case, we will report a case that presented small intestinal perforation and thoracic vertebral fracture caused by a different mechanism.

**Case presentation:**

Previously healthy 48-year-old male presented to the emergency department following falling from 15 feats height. He was a paraplegic with a sensory level at T12. He did not have a clinical feature of spinal shock. He was complaining of epigastric and central abdominal pain and tenderness and was diagnosed to have a proximal Jejunal perforation associated with an unstable fracture of T12 causing spinal compression. Open intestinal repair followed by a posterior spinal exploration and pedicle screw fixation done.

**Clinical discussion:**

Violent injury due to different mechanisms can have similar injuries to Seat belt syndrome. Ultrasonography is used to detect pneumoperitoneum, but the Contrast study is the gold standard to detect visceral injuries. The surgical approach to visceral injury depends on the patient's condition. But the laparoscopic approach has a more favorable postoperative outcome than open access.

**Conclusion:**

Intestinal perforations associated with the neurological deficit are difficult to identify in an initial clinical assessment. Thoracolumbar fractures can associate with small bowel injuries during high-velocity trauma. Early identification and repair of the intestinal injury are important to prevent devastating complications and to improve neurological recovery after spinal surgery.

## Highlights

• Seat belt syndrome is associated with a characteristic set of visceral injuries and spinal fractures.
• Violent injuries caused by a different mechanism can cause similar injuries to Seat belt syndrome.
• Thorough clinical examination with ultrasonography and contrast studies helps to detect visceral injuries.

## 1 Introduction

Seat belt injury was first described in 1965 on a 19-year-old boy following a motor vehicle injury [1]. Seat belt syndrome is associated with multiple visceral injuries and thoracolumbar vertebral fractures [2], [3]. We present a similar injury associated with thoracic spine fractures caused by a different mechanism. Small intestinal perforation is a common scenario during traumatic blunt abdominal injuries [4]. Clinical assessment and the type of radiological investigation are crucial for the correct diagnosis [5]. Small bowel perforations can easily miss during initial clinical and radiological assessment in the background of neurological involvement and lead to devastating complications [4].

## 2 Method

We report this case in line with the updated consensus-based surgical case report (SCARE) guidelines [6].

## 3 Case report

**Fig. 1:**
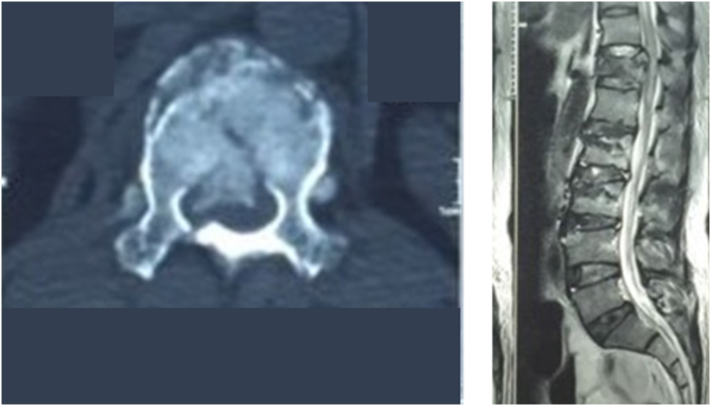
Left image: NCCT scan spine shows T12 vertebral body and right lamina fracture with retropulsion of bony segment into the spinal canal; Right Image: T2 weighted Magnetic resonant image shows compression and edema of the thoracic spinal cord due to retro pulsed bony segment.

A previously healthy, 48-year-old man with an unremarkable drug history and family history was brought to the accident and emergency department following falling from a height of about 15 ft, during which he landed on his back. He complained of severe backache with bilateral lower limb numbness. Neurological examination revealed paraplegia with lower limb muscle power 0/5. The sensory level was detected at T12 Abdominal examination revealed moderate epigastric and central abdominal tenderness with loss of liver dullness. His blood pressure was 110/80 mmHg, his Pulse rate was 80 beats per minute and his Respiratory rate was normal. The Focused assessment with sonography in trauma (FAST) scan was positive for a moderate amount of free fluid, but solid organ injuries were not detected. Noncontrast computer tomography of the spine showed multiple vertebral fractures involving T12, L2, and L3. T12 body and right lamina fracture complicated with retropulsion of a fractured segment causing canal narrowing. L2 and L3 body fractures were undisplaced fractures (Fig. 1). Magnetic resonance imaging study of pan-spine showed a compression fracture of T12 leading to compression myelopathy with cord edema (Fig. 1).

**Fig. 2:**
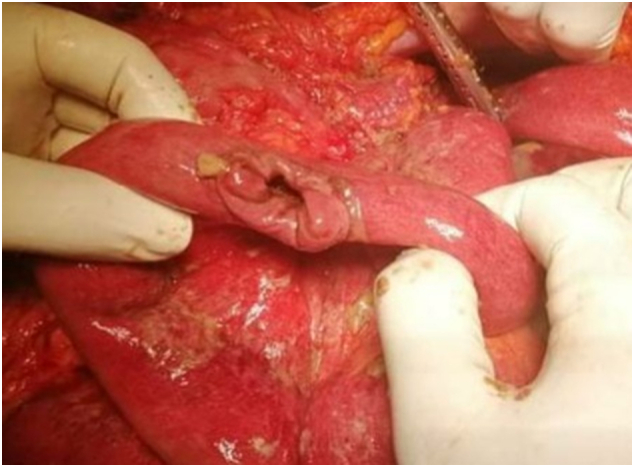
Proximal Jejunal perforation with moderate peritoneal contamination.

Emergency laparotomy was done immediately after admission by the Casualty surgeon of the accident and emergency department.2 cm long proximal jejunal perforation at 12 cm from the duodenojejunal junction in the antimesenteric border has been identified with moderate peritoneal contamination (Fig. 2). The solid-organ injury was not detected. The stomach, Jejunum, and large intestine were normal. Primary repair of the perforation was done with an interrupted Sero-submucosal technique using 3.0 polyglycolic acid sutures.

**Fig. 3:**
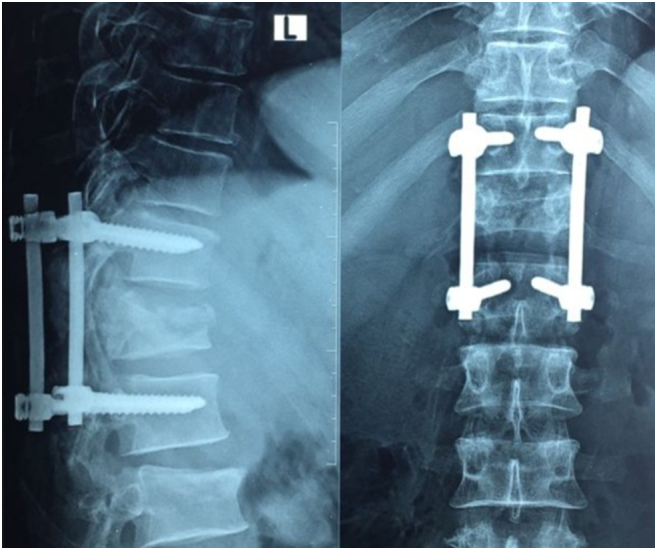
X-ray images of the thoracolumbar spine following short-segment pedicle screw fixation.

The patient was admitted to the ICU following laparotomy. Standard spinal trauma care was provided with strict bed rest, compressive stockings, and nutritional support. Spinal fixation was performed ten days after laparotomy. Exploration of the thoracolumbar spine was done by an Orthopedic Surgeon of the orthopedic and trauma department using a posterior approach. T12 vertebral body and right lamina fracture identified. Spinal decompression and short segment single-level pedicle screw fixation was done (Fig. 3). But Lumbar fractures were not fixed due to extensive dissection and the absence of cord compression.

Rehabilitation was started immediately after spinal fixation. Initially, limb and chest exercises followed by spinal rehabilitation protocol were carried out. Postoperative neurological recovery was not detected below the level of the spinal cord compression and the patient is satisfied with his recovery and mobile in a wheelchair.

## 4 Discussion

In 1965 Howland described a chance fracture in a 19-year-old boy which occurred due to a seat belt following a head-on collision of a car against a large steel pole [1]. Later the name seat belt injury was continuously used for the injuries which have a combination of visceral, vascular, and spinal injury [2], [3]. Commonly associated injuries in seat belt syndrome are Intestinal perforation, Mesenteric tear, aortic injuries, and Thoraco-lumbar vertebral burst fractures [2]. Intestinal perforation is seen in 5–15 % of blunt abdominal injuries [4]. Crushing of the bowel in between the spine and anterior abdominal wall or deceleration injury could be the mechanism [2], [3], [4], [7]. Spinal injuries represent 3 % of overall injuries [8].most of these injuries affect the thoracolumbar region [8]. About 50–60 % of thoracolumbar injuries affect transitional levels(T11-T12) [8].

Visceral perforation and peritonitis are clinically present as epigastric and central abdominal pain and tenderness. Significant peritoneal contamination will cause board-like rigidity of the anterior abdominal wall muscles with features of sepsis. But these symptoms were not apparent in this case due to associated neurological involvement will further attenuate the clinical features of visceral injury [4]. Traumatic intestinal injury is associated with high mortality and morbidity because it is difficult to diagnose and it is associated with severe complications such as bleeding, peritonitis, and sepsis [2], [4].

The sensitivity of the erect abdominal X-ray for large pneumoperitoneum is 50–90% [9]. We didn't do an abdominal X-ray s because positioning was difficult due to spinal cord injury and the FAST scan was readily available with several advantages which make it superior to abdominal X-rays [5]. Supine abdominal radiograph has lower sensitivity for visceral injuries [9]. The FAST scan is widely used to diagnose visceral injuries during trauma. It has a popular diagnostic tool because it is quick to perform, Performs at the bed site, does not expose to radiation [5]. But its sensitivity to detect free fluid is 46.2 % and a negative scan does not exclude the visceral injury [5]. It also has low detection rates for solid organ damage, bowel injury, and mesenteric damage [5]. Therefore if such injury is suspected, going for a laparotomy or performing a further radiological assessment should be decided depending on the patient's condition [5].

A contrast-enhanced CT scan is the gold standard investigation in trauma [5]. It is important to identify the severity of the injury and to decide on the mode of treatment. The presence of free liquid without a sign of a solid organ injury should be considered with great caution because it is associated with a high false-positive value [9], [10]. Therefore decision for surgery should be taken after clinical assessment or repeat CT scan after 6–8 h [10]. Suttle changes in mesentery in CT scan should be correlated with the patient's clinical condition to prevent unnecessary surgeries [10].

The type of the surgical approach should be decided according to the patient's condition [4], [7]. Open surgery or laparoscopic procedure could be used to repair the visceral injury [7]. Emergency laparotomy is indicated for hemodynamically unstable patients without delay. Laparoscopy for diagnostically or therapeutically indicated only for hemodynamically stable patients [4], [7]. Since this patient had moderate free fluid in the FAST scan with a high risk for crashing the blood pressure, Open access was used to enter the abdomen. But perioperative and postoperative outcomes are superior in laparoscopic surgery than in laparotomy [7].

Early reduction of the spine and fixation is associated with the improved neurological function of the patient especially if it is before 72 h from the initial injury [8], [11], [12]. It will further reduce the duration of the ICU and hospital stay [11].Therefor early diagnosis and treatment of the visceral injury is crucial for the improved overall prognosis and early rehabilitation [8], [12]. Approaches to the spine could be anterior, posterior, or combined anterior and posterior [13]. For undisplaced thoracolumbar fracture, the Posterior approach is more preffered [14]. In this case, spinal fixation had to postpone until the patient was recovered from abdominal surgery as positioning for a posterior approach is difficult after laparotomy. But the anterior approach and combined anterior and posterior approaches are associated with intraoperative bleeding and significantly high operative time compared to the posterior aproach [14]. Dural tare is commonly associated with thoracolumbar fracture dislocations [14]. The posterior approach helps to identify the Dural tear and should be repaired to prevent post-operative CSF leak [14].In our case, fracture reduction and single-level above and single-level below fixation were done by pedicle screws. Long segment fixation will give good strength and lower postoperative complications than short segment fixation [14]. Short segment fixation has shown better alignment in the cases affecting thoracic-lumbar junction than lower lumbar cases [15]. But short segment fixation can be used for treating severe injuries as it is associated with less blood loss and decreased operative time [13].

Overall neurological prognosis depends on the severity of the initial injury, Early reduction, and fixation of the spinal fracture, and early rehabilitation [11], [12].

## 5 Conclusion

Traumatic small intestinal injury associated with thoracolumbar fracture can easily be missed during initial clinical assessment and increase mortality and morbidity. Careful clinical assessment combined with radiological investigations will help to identify the visceral injury during trauma. Early identification and repair of intestinal injury will prevent devastating complications. Early intervention for visceral injury is recommended to prevent the delay of spinal reduction and fixation.

## Sources of funding

No institutional or third-party funding sources were used to prepare this case report.

## Ethical approval

This work does not require deliberation by the ethics committee.

## Consent

Informed written consent has been obtained from the patient for the case report and the accompanying images. The written consent is available for review on the request by the editor of the journal.

## Guarantor

Dr. Dilshan Munidasa, Senior Consultant Orthopedic Surgeon and the supervisor of the case report, Department of Trauma and Orthopaedics, National Hospital Colombo, Sri Lanka.

## Research registration

Not applicable.

## Provenance and peer review

Not commissioned, externally peer-reviewed.

## CRediT authorship contribution statement

All authors of this case report are involved in patient assessment, management, data collection, and the preparation of this article. The consultant supervised the management and was involved in the correction and final editing of the article.

## Declaration of competing interest

All authors have declared any competing financial or personal interests which could have influenced their work.
